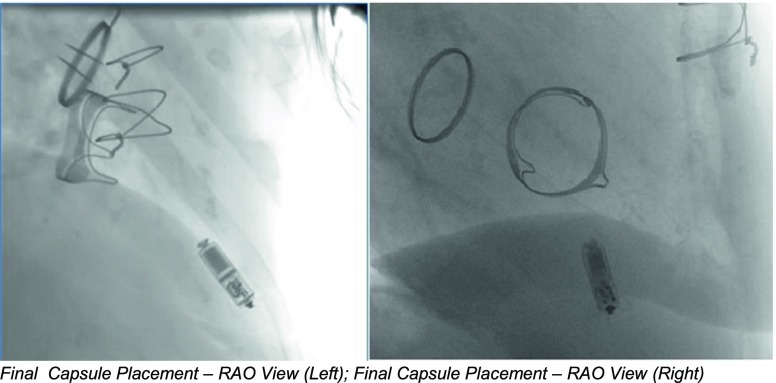# Transcatheter pacemaker implantation in a patient with a bioprosthetic tricuspid valve

**DOI:** 10.1007/s10840-015-0021-5

**Published:** 2015-06-24

**Authors:** Scott A. Kerwin, Mark J. Mayotte, Charles C. Gornick

**Affiliations:** Minneapolis Heart Institute Foundation, Minneapolis, MN USA; Medtronic, Mounds View, Fridley, MN USA

**Keywords:** Pacemaker, Leadless, Bioprosthetic, Tricuspid, Medtronic, Micra

## Background

A 66-year-old white female s/p mitral and tricuspid valve replacement was seen in cardiac consultation and diagnosed with atrial fibrillation and high-degree atrioventricular (AV) block with symptomatic bradycardia, thus pacemaker placement was recommended. For this particular patient, a standard, single-lead implantable pacemaker, while not contraindicated, was not a preferable solution due to the patient’s newly implanted bioprosthetic tricuspid valve and the potential complications transvalvular lead placement may bring [1]. No FDA-approved leadless pacemaker is available in the USA at this time; however, initial safety and feasibility trials have shown comparable results to traditional transvenous leads [2].

## Procedure

The Minneapolis Heart Institute Foundation (MHIF) at Abbott Northwestern Hospital is part of Medtronic’s Micra Transcatheter Pacing FDA IDE trial. The Micra system departs from the traditional design. The pacing capsule is quite small (2.0 g, 0.8 cc) compared with a traditional implantable generator with the added benefit of being leadless.

The contact point for sensing and energy delivery is positioned on one end of the housing and held against the endocardium *via* flexible nitinol tines. The implant procedure is also unique in that it is deployed *via* catheter through the femoral vein rather than the standard pocket formation and access through the axillary veins.

At the start of the procedure, the right femoral vein was entered using the micropuncture technique. Sequential dilatation was performed using an 8 French, 16 French, and then ultimately the large bore 23 French Micra introducer.
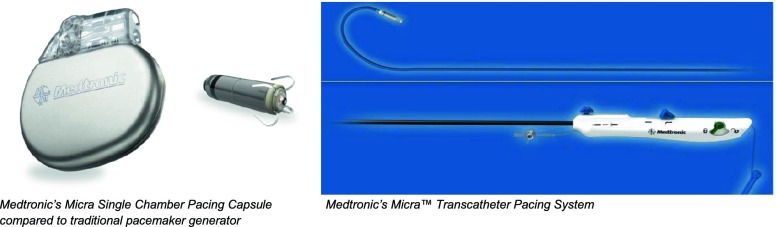


Once the large bore sheath had been placed and advanced over a stiff guidewire to the right atrium, the Micra pacemaker was advanced through this sheath, and using catheter manipulation, directed through the bioprosthetic tricuspid valve toward the apex of the right ventricle. The initial attachment point in the apex while anatomically stable was ultimately unsuccessful due to insufficient electrical measurements. The pacing capsule was retrieved and redeployed in a second spot closely adjacent, which demonstrated excellent sensing and pacing characteristics. Stability was confirmed using the pull and hold test to assure at least two tines were engaged.

After confirming stability, and assessing electrical parameters again, the tether was cut and slowly removed, the delivery catheter and sheath were removed, and the insertion site was closed with a figure 8 stitch. This was the first implant of its kind at this institution. The implant procedure lasted approximately 45 min from initial puncture through closure. The elapsed time was 31 min from the point at which the TPS was inserted into the sheath until the delivery tool was removed.
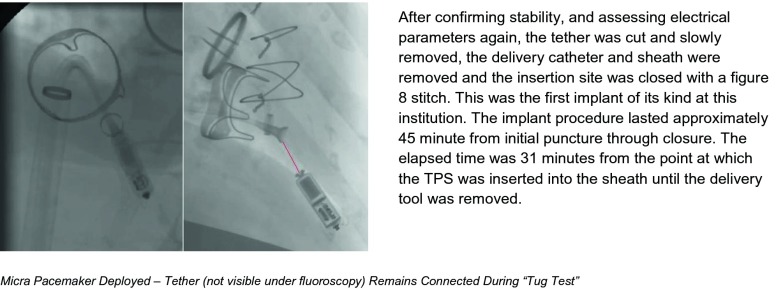


## Performance

Six months post-implant, the patient has had no complications from the procedure or any other cardiovascular-related adverse events. Device data comparing the day of discharge to the 6-month follow-up show acceptable performance and battery longevity:

**Table:** 

Discharge measurements		6-Month measurements	
Vsense	11.5 mV	Vsense	>20.0 mV
Vcapture	0.38 V @ 0.24 ms	Capture	0.38 V @ 0.24 ms
Impedance	650 Ω	Impedance	900 Ω
V-paced %	46.5 %	V-paced %	17.4 %
Battery voltage	3.14 V	Battery voltage	3.10 V
Longevity est.	>13 Years	Longevity est.	>13 Years

Echocardiographic follow-up indicates no significant changes in tricuspid valve performance:

**Table:** 

	TTE—05/2014	TTE—03/2015
TR	TRACE	TRACE
TV mean gradient	4.1 mmHg	3.0 mmHg
TR Vmax	2.5 m/s	2.3 m/s

## Discussion

For our patient, a pacing lead permanently placed through the newly implanted bioprosthetic tricuspid valve is less than ideal as valvular insufficiency could be a concern. Thus, the transcatheter system seemed to have a significant advantage.